# Expression analysis of beta-secretase 1 (BACE1) enzyme in peripheral blood of patients with Alzheimer's disease

**DOI:** 10.22088/cjim.10.3.276

**Published:** 2019

**Authors:** Alireza Vakilian, Javad Masoumi, Saeed Mirzaee, Hossein Khorramdelazad

**Affiliations:** 1 Non Communicable Diseases Research Center, Rafsanjan University of Medical Science, Rafsanjan, Iran; 2 Neurology Department, School of Medicine, Rafsanjan University of Medical Sciences, Rafsanjan, Iran; 3 Molecular Medicine Research Center, Research Institute of Basic Medical Sciences, Rafsanjan University of Medical Sciences, Rafsanjan, Iran; 4 Department of Immunology, School of Medicine, Rafsanjan University of Medical Sciences, Rafsanjan, Iran

**Keywords:** Beta-Site APP-Cleaving Enzyme 1, Alzheimer ’s disease, Biomarker

## Abstract

**Background:**

Recent evidence has indicated that beta-secretase 1 (BACE1) is involved in the production of amyloid beta (Aβ) in patients affected with Alzheimer’s disease (AD). Therefore; the purpose of this study was to measure mRNA and plasma levels of BACE1 in AD patients, as an early diagnosis biomarker for such individuals.

**Methods:**

A total number of thirty AD patients and thirty normal subjects as controls were recriuted in the present study. Plasma levels of BACE1 were then examined via enzyme-linked immunosorbent assay (ELISA) and also mRNA expression of BACE1 in total blood was measured using real-time PCR technique.

**Results:**

The findings revealed a significant difference in gene expression of BACE1 in the peripheral blood of AD patients compared with that in controls (p<0.0001). Additionally, elevated plasma levels of BACE1 were found in AD patients compared with those in normal subjects (p<0.01). Statistical analyses also demonstrated no correlation between expression (mRNA and protein) of BACE1 in both AD patients and controls and age or the results of Mini-Mental State Examination (MMSE) scale (p>0.05).

**Conclusion:**

Given the importance of early diagnosis of AD patients, it was suggested that the measurement of plasma levels and also mRNA expression of BACE1 might be a valuable blood-based biomarker used in preference to other invasive diagnostic methods such as cerebrospinal fluid (CSF) analysis.

Alzheimer’s disease (AD) is known as a progressive neurodegenerative disease, and evidence has indicated that it is the most dominant form of dementia in the elderly (1). Moreover, deposits of extracellular Aβ protein and intracellular neurofibrillary tangles (NFTs) are involved in the pathophysiology of AD through the formation of senile plaques (2). Previous human and experimental studies have also revealed that activity and protein levels of beta-secretase 1 (BACE1) can have a remarkable elevation in the brain tissue and cerebrospinal fluid (CSF) of patients suffering from AD and a positive correlation has been even reported between Aβ protein load and increased levels of BACE1 (3, 4). However, few studies had found that the source of BACE1 could be non-neuronal cells (5). Increased activity of the enzyme and some of the Aβ protein soluble secreted segments in the plasma of AD patients had been also observed (6). Currently; imaging techniques such as magnetic resonance imaging (MRI), positron-emission tomography (PET) or analysis of CSF are utilized for the early diagnosis of AD despite the fact that each method is encountering problems such as high costs, inaccessibility at all locations, and invasiveness (7).

Furthermore; it should be noted that high potential biomarkers, including blood-based biomarkers, MRI biomarkers, PET biomarkers, as well as CSF biomarkers can play an important role in early prediction, screening, and diagnosis of AD patients (8). In this regard, blood-based biomarkers have become especially of utmost significance thanks to their better patient acceptance, cost-effectiveness, and less invasive sampling methods (8). However, the use of these biomarkers in clinical practice has not become routine and there are numerous obscurities in this domain. Therefore, the purpose of this study was to measure mRNA and plasma levels of BACE1 in the peripheral blood of AD patients in comparison with normal subjects considering the evaluation of a novel and profitable blood-based biomarker for the early diagnosis of AD patients. In this study, all of the AD patients were CDR1 and lower grade. Additionally, the enzyme-linked immunosorbent assay (ELISA) kit which was used in this study had a better sensitivity compared to previous studies.

## Methods

***Subjects: ***In this study, a total number of thirty AD patients diagnosed based on diagnostic criteria for AD (National Institute of Neurological Disorders and Stroke-Alzheimer Disease and Related Disorders Association) (9) along with thirty healthy controls were recruited. The AD patients and the control subjects were also matched in terms of variables such as age and gender. Moreover; subjects with schizophrenia, major depressive disorder, mental retardation, substance-related disorder; bipolar disorder, hydrocephalus, cerebrovascular disease, clinically considerable variations in vitamin B12, cranial trauma, visual and auditory disorders (non-correctable), non-compensated hypertension, hypothyroidism, syphilis, diabetes, neoplasia; as well as cardiac, pulmonary, hepatic, and renal diseases were excluded from this study (7). The Mini-Mental State Examination (MMSE) was used as a score for the diagnosis of dementia and also clinical dementia rating (CDR) was applied as the index for the severity of AD symptoms in the patients. These individuals demonstrated cerebral atrophy in cranial MRI or CT scan and no cerebrovascular lesions. It should be noted that CSF was not measured for the diagnosis of the AD patients. All these individuals were also undergoing treatments with one of the cholinesterase inhibitors such as donepezil, rivastigmine, galantamine, or N-methyl D-aspartate receptor antagonist (memantine). This study was approved by the Ethics Committee of Rafsanjan University of Medical Sciences, Iran. In addition, informed consent was obtained from all study subjects or their families in accordance with the Declaration of Helsinki.

***BACE1*** ***Immunoassay: ***The plasma levels of BACE1 were measured using an immunoassay kit (CUSABIO, USA). The measurement procedure was also fulfilled according to the guidelines described in user’s instructions provided by the manufacturer. As observed in the kit’s instructions, the sensitivity of the kit was 0.78 pg/mL. It should be noted that the data were only used when inter- and intra-assays produced scores of CV<15% and CV<5%, respectively.

***Extraction of RNA,*** ***Reverse Transcription,*** ***and*** ***Quantitative Real-Time*** ***PCR: ***The peripheral blood leukocytes were isolated from collected whole blood and the total mRNA content was isolated from the peripheral blood leukocytes using an mRNA extraction kit (Cat.# 9767, Takara, Kyoto, Japan). Briefly; lysis suspension leukocytes were centrifuged at 8,000 Xg at 4^°^C for 2 minutes, then the supernatant was discarded and the cell pellet was re-suspended with 1X PBS, and centrifuged at 8,000 Xg at 4^°^C for 2 minutes again. An appropriate volume of the RL buffer was also added to the cell pellets and the cell lysates were incubated at room temperature for 2 minutes. Next, the lysates were applied to gDNA Eraser Spin Column in the collection tube and subsequently centrifuged at 12,000 rpm for 1 minute. gDNA Eraser Spin Column was also discarded and an equal volume of 70% ethanol was added to the flow-through in the 2 ml collection tube and mixed thoroughly by pipetting it up and down. Furthermore, the mixture was applied to RNA Spin Column in 2-ml collection tube and centrifuged at 12,000 rpm for 1 minute. The flow-through was similarly discarded and 500 μl Buffer RWA was added to RNA Spin Column and subsequently centrifuged at 12,000 rpm for 30 seconds and the flow-through was discarded (this step was repeated). Finally, 350 μl of RWB was added to the RNA Spin Column and centrifuged at 12,000 rpm for 30 seconds.

The cDNA was generated from the yielded total mRNA, employing PrimeScript™ II 1st strand cDNA Synthesis Kit (TaKaRa Bio, Shiga, Japan). The RT-PCR was subsequently performed in a 20 μL solution containing 10 μL SYBR® Premix Ex Taq TM (Takara, Japan), 0.4 μL ROX, 1.5 μL cDNA, and 0.5 μM of each primer, specific for BACE1 and β-actin and up to 20 μL nuclease-free water. The related sequences for primers of the reference and target genes were presented in Table 1. Each separate RT-PCR assay was also completed in triplicate using a thermal cycler ABI Step One Plus® system (Applied Biosystems™, USA). As well, the actin-β was considered as a house-keeping gene (reference gene) aimed at normalization of the amplified signals of the BACE1 gene. Additionally, the relative BACE1 levels were calculated by 2^-∆∆Ct^ wherein ∆Ct = Ct (BACE1)-Ct (Actin-β) and ∆∆Ct = ∆Ct (peripheral blood leukocytes of AD patients)-∆Ct (peripheral blood leukocytes of normal subjects) (10). 

The SPSS stastistics software (Version 18) was used for the statistical analysis of the data. In this resepct, Student’s t-test and Mann-Whitney U test were applied for normally and non-normally distributed data since continuous variables were compared. As well, categorical data were compared through chi-square test. To calculate the correlation coefficient between the scales, Pearson’s correlation coefficient and Spearman’s rank correlation coefficient were used for normally and non-normally distributed data; resepctively. Differences were also considered significant, whereby the p-value was less than 0.05.

**Table 1:** Demonstrates the sequences of primers used in the study

**Gene**	**Forward**	**Reverse**	**Ta**	**Product length**
**Actin β**	*GCGCGGCTACAGCTTCA*	*CTTAATGTCACGCACGATTTCC*	58	55 bp
**BACE1**	*GCCATCTGCGCCCTCTT*	*AGCGCCACTGACACACCAT*	58	55 bp

Ta: annealing temperature

; bp: base per

## Results

***Demographic and Clinical Characteristics: ***A total number of thirty patients affected with AD (12 women and 18 men) and thirty healthy subjects (10 women and 20 men) were recruited in this study. The demographic and the clinical characteristics of these patients were illustrated in Table 2. It should be noted that there was no significant difference between patients and healthy subjects in terms of age (p>0.05) and gender (p>0.05) (Table 2). 

**Table 2:** Clinical characteristics of AD patients and normal subjects

**Gender**	**Male**	**18**	**20**	**NS**
	**Female**	**12**	**10**	
** Age (years)**		71.9±9.3	56.2±11.8	NS
Educational background	Illiteracy	25 (83% )	20 (67% )	NS
	literacy	5 (17%)	10 (33% )	
**MMSE**		14.10±6.8	29.87±5.4	<0.05
**Plasma levels of BACE1 (pg/mL)**		27±7.4	8±2.6	<0.01

NS: non-significant (>0.05)

***Plasma*** ***Level*** ***of BACE1: ***The results of this study showed a significant difference between the plasma levels of BACE1 in patients with AD (27±7.4 pg/mL) compared with those in healthy controls (8±2.6 pg/mL) (p<0.01). Statistical analysis also revealed no correlation between the expression (mRNA and protein) of BACE1 in both AD patients and controls and age or MMSE scale (p>0.05).

**Fig 1:**
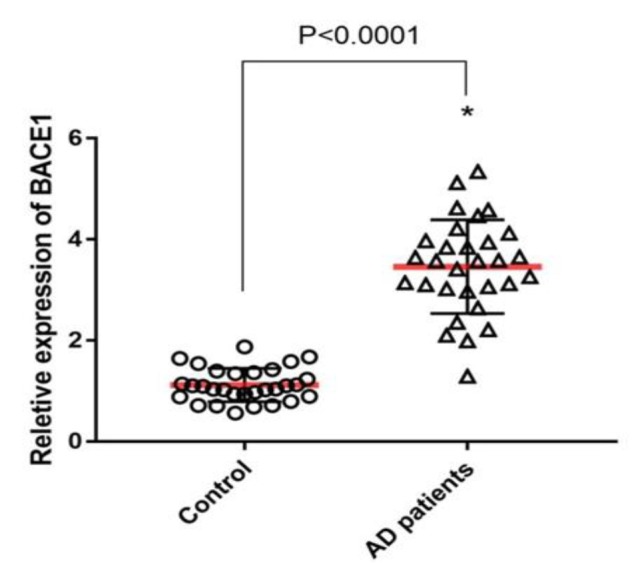
Demonstrates alteration in mRNA level of BACE1 in the peripheral blood of AD patients and normal subjects. Data are presented as mean ± SD, *= significant (P> 0.05)

***BACE1*** ***Gene Expression*** ***(RT-PCR): ***The results of RT-PCR analysis indicated a significant change in the gene expression of BACE1 using peripheral leukocytes on mRNA levels in AD patients compared with those in normal subjects (p<0.0001) (Figure 1).

## Discussion

In this study, the plasma levels of BACE1 in AD patients was examined and then the results were compared with those in healthy subjects. The findings of the present study also showed a significant change in the gene expression of BACE1 in peripheral blood and plasma levels of BACE1 between AD patients and healthy controls. Furthermore, data analysis revealed no correlation between serum levels of BACE1 and clinical symptoms and AD severity. 

Evidence had also shown that amyloid precursor protein (APP) and its proteolysis via β- and γ-secretase enzymes were responsible for amyloidogenesis and generation of Aβ (11). In addition, previous studies had reported that BACE1 was an important β-secretase (12) and animal-based studies had suggested that BACE1 as a major β-secretase, BACE2, and cathepsins might be involved in the formation of amyloid plaques (13, 14). In addition to the brain, the interesting point was that BACE1 could be found in other tissues of the body (15). 

To find about the various interactions between soluble blood proteins and Aβ, measurement of Aβ plasma levels had not been able to provide a profitable diagnostic marker for AD patients (16). Moreover, the plasma levels of Aβ had demonstrated no significant alteration in AD patinets compared with normal subjects (17). Measurment of BACE1 levels in CSF of AD patients had also revealed the possibility of determining the levels of this enzyme in the blood and its compartments (18). Hence, given the few studies conducted on BACE1 measurements in the peripheral blood of AD patients, finding the answer to the question of whether the prepheral blood expression of the BACE1 could be used as a suitable early diagnostic biomarker could be worthwhile.

In this respect Wu et al. reported a remarkable increasing trend in the activity BACE1 in the plasma of AD patients compared with that in normal subjects (6). Furthermore, findings by Manzine et al. highlighted a considerable elevation in the plasma levels of BACE1 in AD patients compared with those in non-AD controls (7). These results were consistent with the findings in the present study, except that in the investigation by Wu et al. in which BACE1 activity was assessed, although the given parameters were closely related in most cases. The results of another study also stated that BACE1 enzyme activity was significantly elevated in the CSF of early-stage AD patients (19). In the present study, all of the AD patients were selected from the CDR stage one or lower and the sample size (n=30) and ELISA kits’ sensitivity (0.78 pg/mL) was improved compared with Manzine’s study (CDR1 patients=7 and ELISA kits’ sensitivity=1.0 pg/mL). The expression of BACE1 in peripheral blood mononuclear cell (PBMC) had been the subject of a few investigations and the relevant reports had shown lower amounts of BACE1 mRNA in PBMC compared with those in the brain (20). Contrary to the results of this study, Manzine et al. inferred that there was no significant change in the mRNA level of BACE1 in the peripheral blood of AD patients compared with that in normal subjects (7). Recently, in line with the findings of the present study, Feng et al. have reported that the plasma long non-coding RNA (LncRNA) BACE1 level of patients affected with AD was significantly higher than that of healthy controls (21). The discrepancies in the mentioned investigations might be due to differences in their methodologies; so, the type of biologic specimen, techniques, kits, sample size, and demographic characteristics of subjects, adminstered drugs (type and dose), as well as severity of the disease could have affected the outcomes. 

Considering all the variations influencing blood BACE1 levels and activity, it could be concluded that mRNA and protein levels of BACE1 might be an early blood-based diagnostic tool for AD patients. Nonetheless, further studies with larger sample size and different methods such as western blotting or immunohistochemistry on brain tissue samples in transgenic mouse model of AD as well as measurement and comparison of the levels of BACE1 in CSF, plasma, and PBMCs of AD patients were needed to confirm the role of this enzyme in the patogenesis of AD. In this way, the potential of BACE1 was highlighted as an early diagnostic biomarker in AD patients.
